# Thermomechanical and Morphological Studies of CFRP Tested in Different Environmental Conditions

**DOI:** 10.3390/ma12010063

**Published:** 2018-12-25

**Authors:** Claudia Barile, Caterina Casavola, Paramsamy Kannan Vimalathithan, Marco Pugliese, Vincenzo Maiorano

**Affiliations:** 1 Dipartimento di Meccanica, Matematica e Management, Politecnico di Bari, Viale Japigia 182, 70126 Bari, Italy; casavola@poliba.it (C.C.); pk.vimalathithan@poliba.it (P.K.V.); 2 Istituto di Nanotecnologia—CNR Nanotec—c/o Campus Ecotekne, strada provinciale Lecce-Monteroni, 73100 Lecce, Italy; marco.pugliese@nanotec.cnr.it (M.P.); vincenzo.maiorano@nanotec.cnr.it (V.M.)

**Keywords:** CFRP, TGA, activation energy, environmental tests, optimum working temperature, SEM

## Abstract

The present work describes the mechanical characterization combined with the thermal degradation kinetics of Carbon Fiber Reinforced Polymers (CFRP). The thermal degradation kinetics of CFRP have never been studied in the past. In that regard, the present work focuses on studying the thermal degradation kinetics of CFRP tested mechanically at different environmental conditions. Tensile tests were performed on the specimens with different lay-ups at room temperature, elevated temperature (71 °C), and cryogenic conditions (−54 °C), and the same specimens were used for thermal degradation kinetic studies. Mechanical tests show different responses respect to the different environmental conditions and different fibers orientation. On the other hand, the thermogravimetric results, mass loss, and derivative mass loss, show no significant difference in the degradation of CFRP tested at different temperatures. However, the thermal degradation kinetics shows more insight into the degradation pattern of the materials. The activation energy of degradation shows that the degradation of materials subjected to elevated conditions increases rapidly in the later stages of degradation, suggesting the formation of high char yield. The varying activation energy has been related to different degradation mechanisms. Lastly, the morphology of the materials was studied under SEM to understand the structural change in the material after tested in different weather conditions.

## 1. Introduction

In recent years, Carbon Fiber Reinforced Polymers (CFRP) have been widely used in aerospace applications. They are advantageous over their metallic alloy counterparts owing to their mechanical properties and high strength to weight ratio. They also have a low thermal expansion, high fatigue resistance, and good corrosion resistance under different environmental conditions [1]. Moreover, most of the above-mentioned properties can be tailored for specific applications. The usage of CFRP has increased exponentially since it found itself in the load carrying parts of Boeing 787, Airbus 350, and F-35. 

The properties of the CFRPs are based on both the polymers and the carbon fibers used as the reinforcement. In aerospace and naval application, the epoxy resins are commonly used as the matrix for preparing CFRP prepreg (carbon fibers have excellent mechanical properties). The reinforcement is crucial in determining the strength of CFRP. Carbon fibers have superior Young’s modulus when compared to the E-Glass fibers which have Young’s modulus of 25 GPa. They have an ultimate tensile strength and tensile modulus which are comparable with steel and aluminum and can exceed the properties of steel and aluminum based on their preparation and surface treatments. The nominal requirement of carbon fibers has increased from 111,785 tons in 2012 to 156,845 tons in 2016. It has increased even further in the present year and anticipated to exceed 290,000 tons by 2024. However, the production and usage of carbon fiber have been facing sever cost constraints [2,3].

European Union’s waste framework has regulated the management of composite waste. It aims to reduce the waste disposal and incineration of the composite waste; reusing, recycling, and recovering of useful materials from the composite waste [3].

Over the past year, pyrolysis has been employed in recovering the carbon fiber from the CFRP waste [1,4,5,6]. The CFRP wastes are pyrolyzed at 400 to 700 °C in an inert atmosphere to break down the epoxy macromolecule into smaller molecules and evaporating them to recover the residue carbon fiber. This is a high charring process which yields plenty of char from which the carbon fibers have to be recovered [1,7].

Although the degradation of epoxy resins and their composites has been studied extensively over the years, some of the key parameters have been ignored in these studies. The CFRPs, which are used in aerospace industries, are subjected to various environmental deteriorations. For instance, the temperature closer to the parts of an aerospace engine can increase up to 71 °C and at the same time, at the high altitude, the temperature of some parts of the aircraft can reach as low as −54 °C This completely changes when the aircraft is not at function [8,9]. The CFRPs used in these structures maybe subjected to freeze-thaw conditions. There is a possibility that these altering environmental conditions can affect the thermal characteristics of the CFRPs. Consequently, it would be of great significance to know the thermal characteristics of the CFRP subjected to varying environmental conditions.

The present study aims in studying the thermomechanical characteristics of CFRP tested under three different environmental conditions [10]. Mechanical properties have been collected with respect to Young’s modulus and tensile strength. The thermal kinetic parameters, the activation energy, pre-exponential factor, and the optimum working temperature of the material have been studied using isoconversional methods. 

The activation energy refers to the energy absorbed in the process of breaking inactive macromolecule into smaller molecules to activate the degradation process. It is the energy required to overcome the energy barrier for the reaction to occur. The pre-exponential factor is the frequency at which the reaction is occurring. The optimum working temperature is a quantifying parameter to estimate the temperature at which the material begins to lose its properties over a specific period of time. These parameters can be studied using the results from thermogravimetric (TG) analysis when the degradation is a result of chemical processes. 

In the present study, the specimens recovered after tensile testing in different environmental conditions were taken. The TG analysis was performed in an inert (nitrogen) atmosphere and the thermal kinetic analysis was studied. The microstructure of the specimen subjected to testing in different conditions was also analyzed under the scanning electron microscope (SEM).

## 2. Materials and Methods 

The CFRPs were prepared using Resin Film Infusion (RFI) at 135 °C and 1.5 bar pressure for 2 h [10,11]. The RFI process is faster, cheaper, and capable of producing more complex components with better dimensional tolerances than the more traditional methods. The stitching process, instead, should improve the strength normal to the fibers direction, reducing delamination effects and buckling phenomena. The material tested is composed of carbon fibers and epoxy matrix and it has been produced in sixteen tiles of specific configurations. The base epoxy used for preparing the prepreg is a blend of bisphenol-A type epoxy, novolac type epoxy, and tetraglycidyl diamino diphenyl methane (TGDDM) epoxy, while diaminodiphenyl sulfone is used as the hardener [12,13]. The average resin content in the CFRP prepreg is ~38%. The carbon fibers used as the reinforcement have an average fiber density of 1.79 gm/cm^3^ and fiber diameter of 5 µm. Three kinds of fibers orientation have been mechanically tested: 33/33/33, 40/40/20, and 100/0/0. Each number of the series refers to the percentage of fibers oriented along three directions (0°/±45°/90°) with respect to the zero lamina, which is the fibers’ direction of the top surface layer. 

Three different specimen groups were considered for this study. They differ one from each other for fiber orientation along the three main directions 0°/±45°/90°, and the percentage resin content. The specimens were subjected to tensile tests under three different environmental conditions: room temperature 24 °C (RT), hot/wet conditions at high temperature 71 °C (H/W), and cryogenic conditions at −54 °C (CT). According to the standard ASTM D 3039, the specimens tested had a rectangular shape 250 mm long, 25 mm wide, and 2.5 mm thick. The results of the CFRP tested are presented in the subsequent sections. They refer to mean value of three replications of the same mechanical test at the same environmental condition. Beyond this, a total of 163 samples were subjected to a compression test, open hole compression, and a filled hole compression test and their results are presented in the previous research work [10]. The details of the resin content, fiber orientation, number of plies, testing conditions, and the specimen denominations are presented in Table 1. 

The specimens A2, B2, and C2 simulate the real conditions close to the aircraft engine by wetting treatment. The procedure involves exposing the specimens to 85% relative humidity and 66 °C temperature until an equilibrium weight gain was obtained. Immediately following that, the specimens were sealed in a bag with a cotton towel until testing. The tensile test was performed in an environmental chamber by increasing the temperature from room temperature to 71 °C at a rate of 5 °C/min.

Similarly, for testing the specimens A3, B3, and C3, the chamber temperature was decreased from room temperature to −54 °C using nitrogen. Also, in this case, the temperature rate was 5 °C/min, starting from room temperature to the test temperature. The specimens for TG analysis were collected from the broken tensile tested specimens. The specimens were held firmly in a vice and using end mill cutter, a segment of the specimen was collected approximately 5 mm from the region of breakage. Approximately 20 mg of specimens were taken for TG analysis from the cut segment [14].

### 2.1. Mechanical Tests

According to the ASTM D3039, the mechanical tests were performed in displacement-controlled mode at a crosshead displacement speed of 2 mm/min, on a servo-hydraulic testing machine (INSTRON 1320, capacity load 200 kN, Norwood, MA, USA). The testing machine has a load capacity of 100 kN. The tensile load was applied along to the longitudinal axis of the specimens that were cut along three different directions (0°, 45°, and 90°) with respect to zero lamina. The ones used in this part of the research were of the 0° orientation. Electrical strain gauges were attached to the midsection of the specimen to evaluate the strain under loading conditions. For all the specimens tested, the ultimate tensile strength σ_u_ and Young’s modulus were recorded by directly coupling the tensile testing machine and strain gauge to the data acquisition module. The Young’s modulus and the ultimate tensile strength are calculated according to the standard [15].

### 2.2. Thermogravimetric Analysis (TG)

According to the recommendations of the International Confederation of Thermal Analysis and Calorimetry (ICTAC), for thermal kinetic analysis, the TG analysis of materials must be carried out at least in four different heating rate programs [16]. The TG analysis was carried out in a thermogravimetric apparatus (TGA model Q50 supplied by TA Instruments, New Castle, DE, USA). Samples from each specimen of small granules, approximately 8 to 20 mg were taken for the test. The specimens were kept at an isothermal temperature of 50 °C for 5 min before applying the specific heating rate. The entire test was carried out in a high purity nitrogen atmosphere with a constant flow rate of 100 mL/min from 50 to 1000 °C. The test was repeated for 4 different heating rates (β), 10, 15, 20, and 25 °C/min. The data obtained from the TG analysis were used to estimate the thermal kinetic parameters, the activation energy, and pre-exponential factor.

### 2.3. SEM Analysis

The morphology of the mechanically tested material in different environmental conditions was characterized under scanning electron microscopy (SEM) (Ziess EVO MA-10 MA, Oberkochen, Germany). The test was conducted in a high vacuum and the secondary electrons were analyzed to understand the surface morphology of the material after failure. The specimens, after the mechanical failure, were held in a vice and approximately 5 mm from the region of damage, a segment of specimen was cut using an end-mill cutter and analyzed by the SEM. 

### 2.4. Theoretical 

Various methods have been employed over the past few decades to understand the thermal kinetic behavior of the material [16]. Nonetheless, all these materials originate from the general isothermal solid-state transformation relation, Equation (1).

(1) dαdt=K(T)f(α)

where, dα is the change in conversion, dt is the change in time, *K(T)* is the reaction rate, and f(α) is the reaction model. However, the isothermal equation makes it tedious and time consuming to estimate the kinetic parameters. Thus, it was recommended to follow non-isothermal conditions for evaluating the kinetic parameters, with different heating rates (β).

(2) β=dTdt,

where *dT* is the change in temperature. The temperature dependence, *K(T)*, of the solid-state transformation process is generally parameterized through Arrhenius equation, Equation (3).

(3) K(T)=Aexp(−ERT)

where *A* is the pre-exponential factor, *E* is the activation energy, and *R* is the gas constant.

Over the past three decades, several critical comments were raised on whether the heterogeneous reaction can be bound by the Arrhenius equations [17]. After numerous experimental campaigns supported by numerical evidences, a consensus was made that the heterogeneous reactions follow the Arrhenius parameterization and can be used for thermal kinetic analysis, but the reaction does not proceed through a single mechanism.

The introduction of Arrhenius equation in the solid-state kinetics has given rise to the complexity in parameterizing the activation energy (*E*) and pre-exponential factor (*A*).

(4) βdαdT=Aexp(−ERT)f(α)

This equation forms the basis of the isoconversional principle and has ever since been used to estimate the kinetic parameters, the activation energy, pre-exponential factor, and reaction model.

#### 2.4.1. Activation Energy

The isoconversional principle states that reaction rate at the constant extent of conversion is only a function of temperature. A number of isoconversional methods have been proposed over the years for estimating the kinetic parameters. The accuracies and reliability of the different methods have been debated and validated with experimental results, which can be found elsewhere [16,17].

In the present work, the activation energy is estimated through the C-KAS (Corrected KAS) method, which is the modified iterative version of the classical KAS method. The C-KAS method eliminates the systematic errors arise in the KAS method [18,19]. The two methods were used to provide a perspective on the evaluation of activation energy in different ways.

The general equation of solid-state transformation, Equation (4) is integrated with respect to *dT* and rearranged by assuming x=E/RT.

(5) g(α)=∫0αdαf(α)=Aβ∫0Texp(−ERT)dT

(6) g(α)=AEβR∫x∞exp(−x)x2dx=AEβRp(x)

where, $\int_{x}^{\infty }exp\left(-x\right)/x^{2}dx=p\left(x\right)$. To solve *p*(*x*), number of numerical approximations (called temperature integrals) has been supplied by numerous researchers. The KAS method uses the temperature integral proposed by Coats-Redfern [20,21].

(7) p(x)=exp(−x)x2(1−2x)

Now, by taking the natural logarithm of Equation (6) and replacing *p(x)* with Equation (7), eliminating the asymptotic approximation, the following equation can be obtained.

(8) lnβiTi2=lnARg(α)−ERTi

where, *i* is the number of the heating program and g(α) is the integral form of the reaction model. By taking the slope between the left-hand side of Equation (8) and its right-hand side, the activation energy for any specific conversion (α) can be estimated. This is the procedure that follows estimating activation energy through KAS method.

The C-KAS method proposed by Farjas [22] has inserted a term ε in Equation (8) of KAS method. This new iteration procedure has eliminated the systematic errors that arise in estimating activation energy through the classical KAS method. The term ε can be expressed as follows

(9) ε=p(xi)exp(x)/x2

where, $x_{i}=E/R\bar{T}$ and $\bar{T}$ is the average temperature over which the conversion α lies within. Farjas modified the KAS equation as follows

(10) lnβiTi2−lnε=lnARg(α)−ERTi

Now by iterating the linear equation for each conversion, the activation energy can be estimated. In the present paper, the activation energy for all the materials tested is estimated using C-KAS method.

#### 2.4.2. Pre-Exponential Factor

The frequency of the reaction occurrences can be parameterized by the pre-exponential factor. Although there is a number of approaches in estimating the pre-exponential factor, a simple and recently developed approach has been used in this study [23,24]. It is based on the isoconversional principle that the pre-exponential factor is essentially a parameter of temperature at each conversion and is directly proportional to the activation energy.

(11) A=[Eg(α)R]exp(INT)

where, INT is the intercept obtained from the slope of Equation (10), the C-KAS method.

#### 2.4.3. Optimum Working Temperature

The optimum working temperature is the temperature at which the material can perform for a specific time period before losing its integrity to function. The basis of predicting the optimum working temperature was proposed by Toop [25] in 1971, nonetheless, the concept has been constantly misused ever since. In this work, the rigorous numerical solution provided by Toop has been used to estimate the optimum working temperature of the materials.

(12) lntf=ERTα+ln[Ep(x)βR]

where $t_{f}$ is the thermal lifetime for which the optimum working temperature to be estimated, *p*(*x*) is calculated from Equation (7) and β is the heating rate in °C/h and $T_{\alpha }$ is the optimum working temperature.

## 3. Results and Discussions

### 3.1. Mechanical Tests

Values of stress and strain have been recorded during all experimental tests. At least three replications were performed for each lay-up. The mean value of ultimate tensile strength and Young’s modulus for the specimens tested at the three different environmental conditions were presented in Table 2.

It should be noted that all the three specimen groups are not of the same configuration when it comes to fiber orientation, resin percentage, and number of plies (Table 1). Suffice to say that the mechanical properties of the specimen groups vary from one another. The same was observed during the investigation as well. However, the ultimate tensile strength of specimens tested in cryogenic condition (A3, B3, and C3) is relatively lower than the specimens tested in RT and H/W, in their respective groups. Specimens A3, B3, and C3 have an ultimate tensile strength of 748 MPa, 509 MPa, and 767 MPa, respectively. Since these specimens are tested in cryogenic conditions, the brittleness probably increased, resulting in lower ultimate tensile strength and the highest recorded modulus of elasticity in this study—approximately 2-fold greater than the ones measured at H/W and RT. The evidence for the brittleness in specimen A3, B3, and C3 are provided using SEM images in Section 3.6.

**Figure 1:**
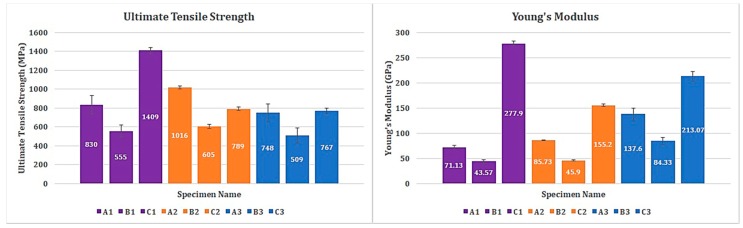
Ultimate tensile strength and Young’s modulus of all specimens tested.

Of the specimens tested in H/W conditions, A2 and B2 have the highest ultimate tensile strength among their respective groups. Nonetheless, specimen C2 has significantly lower tensile strength than C1, which is tested in RT condition. The specific reason for this phenomenon could be explained referring to the unidirectional fiber orientation of C1 specimens. Perhaps, this might have influenced the unexpected change in the ultimate tensile strength. Anyway, it is evident that the majority of the tensile load is transferred directly to the fibers. Since carbon fibers have higher thermal stability and coefficient of expansion than the epoxy matrix, the mechanical properties of the specimen group C is superior when compared to the other two groups (Figure 1).

More detailed information on the mechanical properties and the results can be found in the author’s previous research, where more details on mechanical results of open hole compressive, open hole tensile, and compressive test specimens can be found [10].

### 3.2. Thermogravimetric Analysis

**Figure 2:**
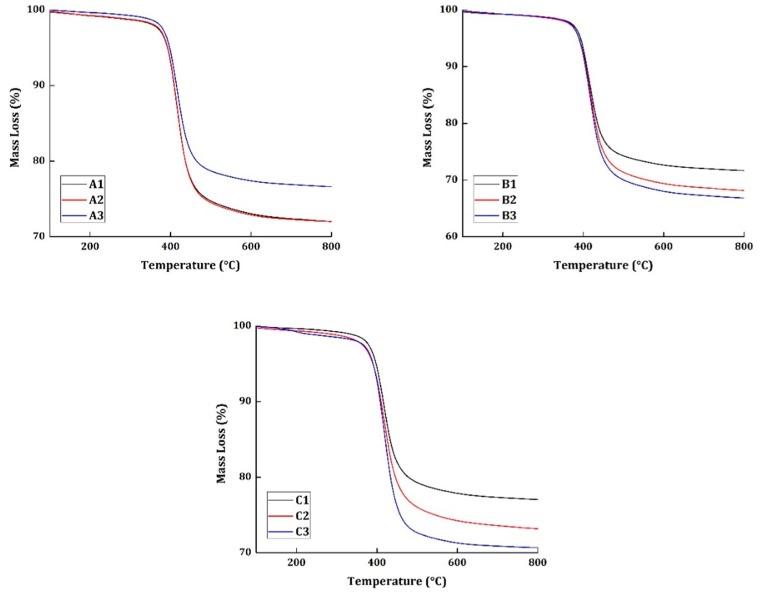
TG results of all the specimens tested at 20 °C/min heating rate.

**Figure 3:**
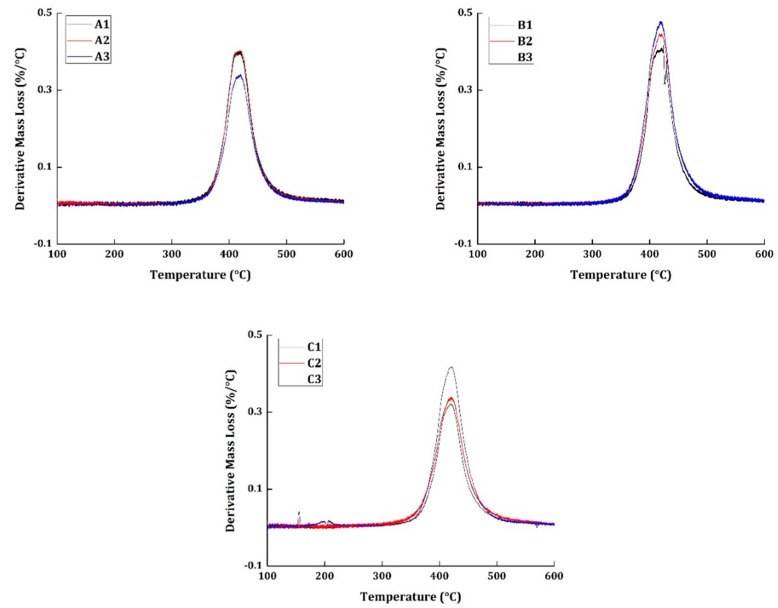
Derivative mass loss results of all the specimens tested at 20 °C/min heating rate.

The thermogravimetric analysis was taken at four different heating rates (10, 15, 20, and 25 °C/min) for all the materials. The basis of thermal kinetic parameter estimation can be done only with different heating rate programs. Figure 2 and Figure 3 show the TG and derivative mass loss results, respectively, of all the materials at the heating rate of 20 °C/min.

In Figure 2, more than 65% of all materials tested have a char residue at 800 °C. This can be attributed to three reasons. The material taken for the study has an average resin content of 38% and, generally, epoxy systems degrade completely at 800 °C. Moreover, the CFRP does not degrade completely and can result in producing its own residue. Perhaps, this is one reason why the char is accounted for more than 65%. The second reason is that it has been reported in previous research works and has been proved evidentially that the vacuum pyrolysis of CFRP is a high charring process [1]. From these chars, the CFRP can be recovered up to 85%. The third reason: it is a very well-known phenomenon that when the material is thermally degraded at higher heating rates, it produces excessive char. Thus, these three possible reasons can be directly related to the high char content in the materials at 800 °C.

Figure 3 shows the derivative mass loss of all the specimens tested at 20 °C/min heating rate program. Epoxy systems, in general, are known to have excellent corrosion, abrasion, and chemical resistance. However, their water resistance is not superior when compared to the other commercially used polymers. The specimens A2, B2, and C2 were subjected to wetting treatment, by exposing them to a relative humidity of 85%. The presence of moisture in the specimen normally would produce a peak around 150 °C regions in the derivative mass loss curves. In the presented results, none of the specimens have shown any peaks around the 150 °C regions to show the evidence for the presence of moisture. It should also be noted that the mechanical tests were conducted for specimens A2, B2, and C2 at an elevated temperature of 71 °C. The moisture, perhaps, had evaporated during the course of testing conditions. 

The degradation commences in all the materials at 310 °C and ends around 550 °C. The presence of carbon fiber is responsible for having the end set temperature of degradation to a higher temperature region. Similarly, maximum degradation occurs at 415 °C. Although all the materials subjected to different environmental conditions show similar TG and derivative mass loss results, it cannot be concluded that degradation occurred in a similar pattern because the degradation of neat epoxy resin commences at 250 °C and has the maximum degradation at 375 °C. 

The charring of the carbon fibers has shifted the onset temperature of degradation to a higher temperature region. Thus, is quite difficult to elucidate the degradation of a CFRP system simply through TG and derivate mass loss date. Accordingly, the thermal kinetics of the materials were studied, and the results are presented in the subsequent sections.

### 3.3. Activation Energy

**Figure 4:**
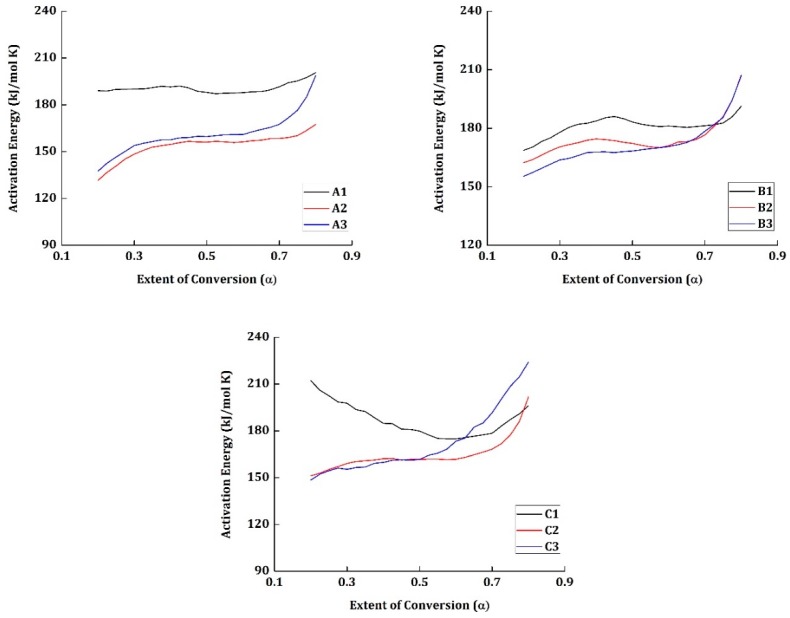
Activation energy vs. extent of conversion for all the materials.

The activation energy for all the materials tested are estimated using an iterative approach (C-KAS method) and is presented in Figure 4.

The specimens which were tested mechanically at room temperature (A1, B1, and C1) have the highest activation energy at the initial conversion *α* = 0.2. The materials A1 and B1 have relatively higher activation energy than the materials in their respective groups and have increased with the increase in conversion. Nonetheless, the material C1 shows a different pattern. At the initial stage of conversion *α* = 0.2, C1 has an activation energy of 210 kJ/mol K but decreases gradually to 178 kJ/mol K at *α* = 0.525; from there, it increases again to reach 196 kJ/mol K at *α* = 0.8.

Despite the materials A1, B1, and C1 being tested mechanically under the same temperature conditions, their degradation activation energy values vary from one another. The presence of the epoxy group in the resin provides more compatibility for the interfacial reaction with the carbon fibers reinforced in it. Moreover, the resin content cannot be guaranteed to be the exact value in the segment of the material taken for TG testing. Thus, the influence of the weight percentage of carbon fiber in the material taken for testing and the interfacial reaction between the epoxy groups and the carbon fibers are responsible for the varying activation energy between materials A1, B1, and C1. In specimen C1, the presence of carbon fiber content must have been more than expected and has a direct impact on the increased activation energy at the initial stages (say *α* = 0.2). 

It has been reported that the vacuum pyrolysis is an accelerated charring process, and the presence of excess char must have resulted in providing a thermal barrier for the volatile products to elapse. This might be the reason why the activation energy has increased from 178 kJ/mol K to 196 kJ/mol K in the material C1. Thus, the highest activation energy cannot be attributed to the different environmental conditions the materials were tested, but to the weight percentile of the epoxy resin and the carbon fiber content. 

Generally, in epoxy systems, the homolytic scission of the C–O and C–N bonds at the initial stages of degradation (at regions *α* < 0.2) influences the physical structure and relaxes the bonds but does not have any significant effect on the mass loss [26,27,28,29,30,31].

The activation energy for most of the materials at the initial stage—*α* = 0.2—lies within 125 to 150 kJ/mol K. The weakest point in the epoxy resin chain is the region where the concentrations of O–CH_2_ and C–N [29]. The activation energy to break the energy barrier to initiate the degradation in these weakest regions is relatively low. Another reason is the high concentration of hydroxyl group, which can lead to the intermolecular hydrogen bonding, promoting dehydration. This shows that the reaction is more facile at the initial stages. Moreover, the degradation is also initiated by C–C bond scission in the chain. The cumulative energy required for these reactions to occur is generally low. This can be related to why the activation energy is low at the initial stages of the degradation. 

**Figure 5:**
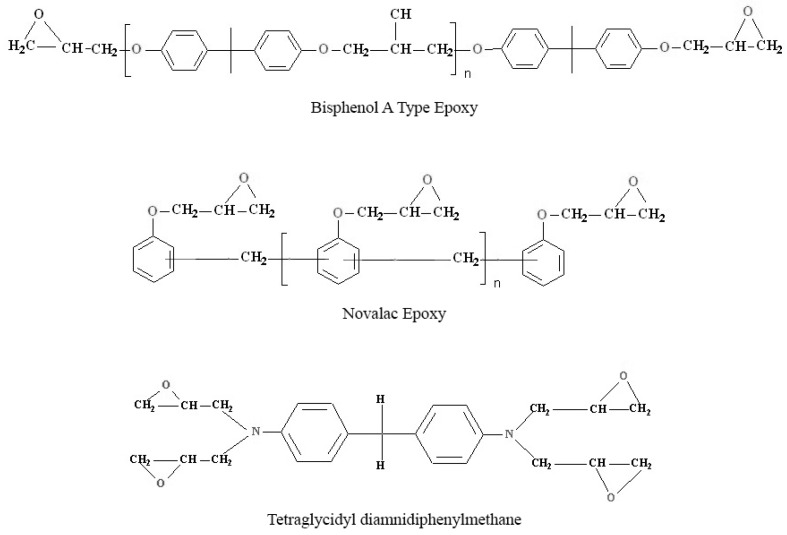
Structure of epoxy system used in this study.

However, following these reactions, the unsaturation of molecules in the system increases the stiffness of the polymer chain. There is also a possibility for the formation of high polyaromatic hydrocarbons (Figure 5). This results in the embrittlement of the polymer chain and the high concentration of the aromatic chains, which are probably the reasons why the activation energy increases gradually in the later stages of conversion.

In the materials, A2, B2, and C2, the average activation energy of conversion is slightly less than the materials A3, B3, and C3, respectively. Especially, in the later regions of conversion (from *α* = 0.6 ÷ 0.8), the activation energy of materials A3, B3, and C3 have increased rapidly when compared to their counterparts. 

The materials A2, B2, and C2 were tested mechanically at 71 °C and the temperature of the system was increased from room temperature to the necessary condition at the rate of 5 °C/min. This probably relaxed the polymer chain which reduced the requirement of activation energy marginally during degradation. Moreover, the materials A3, B3, and C3 were tested at −54 °C and the cryogenic condition probably increased the brittleness of the specimen. The embrittlement of the polymer chains mostly results in the increase of activation energy in the later stages of conversion.

### 3.4. Pre-Exponential Factor

The frequency of collision of molecules during the degradation process can be parametrized by the pre-exponential factor. The pre-exponential factor is directly proportional to the activation energy since it is a parameter of temperature for a specific conversion. The pre-exponential factor was estimated for all the materials tested and is provided in Table 3. 

Similar to the activation energy, the pre-exponential factor also follows the same pattern, except in material group C. As mentioned earlier, the probable reason is the increased weight percentage of carbon fibers in the segment of the material taken for testing. The high concentration of carbon fiber forms an intricate path for the volatile content during the final changes of degradation. This results in accelerating the charring on the outer surface of the specimen resulting in high lnA values. The material C1 has lnA value of 36.21 at *α* = 0.2 and it has increased to 50.94 at *α* = 0.8. However, the pre-exponential factor of specimen C2 is close to material A2 and B2. On the other hand, the same observation can be found between the materials A3, B3, and C3. The material C3 has a significantly higher value of pre-exponential factor when compared to B2 and C2. 

Upon observing the TG and derivative mass loss results, the reaction complexity and the variation in the char formation or the reaction mechanism could not be discriminated between the different materials. The thermal degradation kinetics is the appropriate tool to explain the reaction complexity and the reason for the excessive char residue during thermal degradation. 

Nonetheless, the evidence for the rapid char formation can be quantitatively provided by only by analyzing the volatile compounds liberated during the thermal degradation process. A few researchers have studied the volatile products of degrading CFRP using gas chromatography. The scope for the future of this research work is to expand the degradation kinetics to compare and relate it with the gas chromatography results.

### 3.5. Optimum Working Temperature

The optimum working temperature of all the materials at different lifetime requirements was estimated using the mathematical solution provided by Toop [24] (as in Equation (12)) and has been shown in Table 4. As expected, the material C1 has the maximum working temperature of 170 °C for 20,000 h, 314 °C for 1 h, and so on (Table 4). This is the numerical evidence to show the high carbon fiber content in the material C1, which also contributed to high activation energy and lnA values.

It can also be observed that the materials tested mechanically at elevated temperature (A2, B2, and C2), prior to the TG analysis, has the lowest working temperature. For instance, material A2 can work at 115 °C for 20,000 h when compared to A1 and A3. Similarly, material B2 can work at 134 °C for 20,000 h, compared to B1 and B3 which can operate at 146 °C and 142 °C, respectively. The same observation can be seen in C2 as well. This shows that the materials subjected to high temperature mechanical testing has relaxed the stiffness of the molecular chain, leading to the reduction of optimum working temperature. Nonetheless, the material A3 has quite similar working temperature as A2, 116 °C. This can be attributed to the inadequate resin content in the sample taken for TGA analysis. In Figure 2, it can be seen that the char residue of A3 is almost close to 80%, proving the same. 

At the same time, the materials tested in cryogenic conditions (A3, B3, and C3) also have lower working temperatures when compared to the materials tested in room temperature (A1, B1, and C1). Thus, it is quite evident from the results that environmental conditions not only affect the mechanical properties of a material but also play a significant role in the thermal degradation properties. The materials tested in both the elevated temperature and cryogenic condition have deteriorated, even if marginally, the thermal properties of the material.

### 3.6. SEM Analysis Results

**Figure 6:**
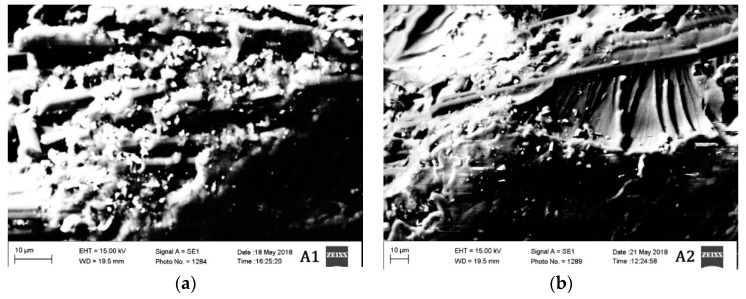

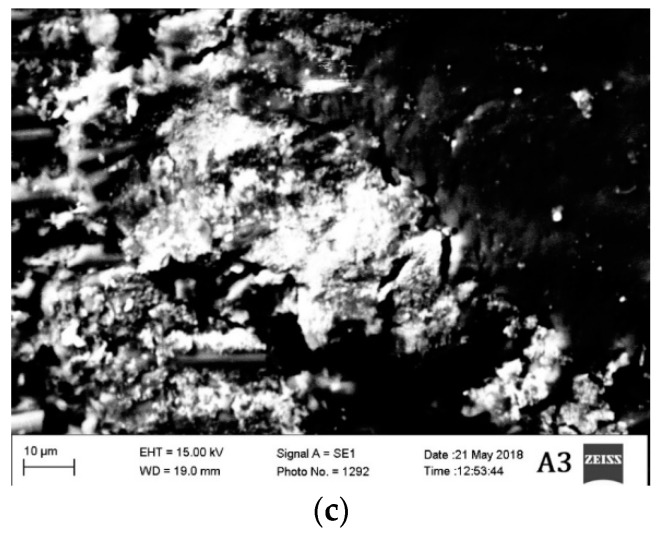
SEM Images of materials (**a**) A1, (**b**) A2, and (**c**)A3.

**Figure 7:**
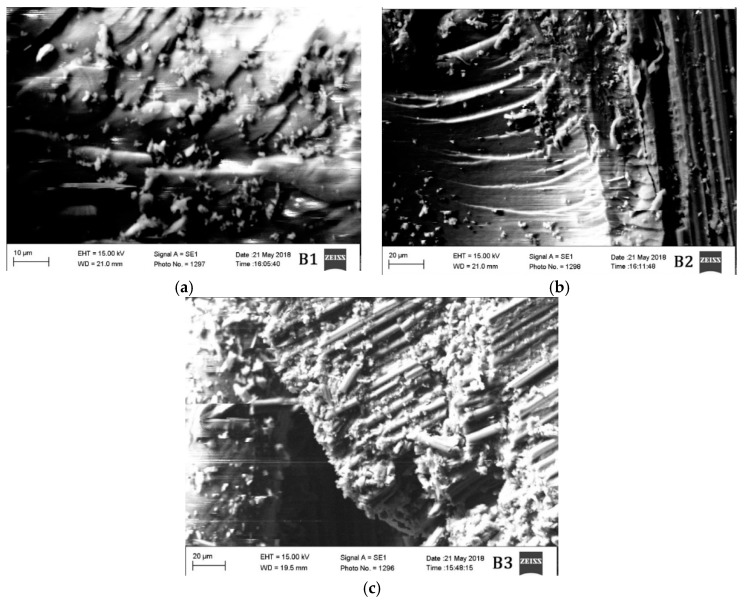
SEM images of materials (**a**) B1, (**b**) B2, and (**c**) B3.

The morphological characteristics of the materials tested in different conditions are also evaluated. Two material groups A and B are studied in high vacuum under scanning electron microscopy (SEM) and the results are provided in Figure 6 and Figure 7.

The segment of material taken approximately from 5 mm from the region of damage after mechanical testing was taken for SEM analysis. Microcracks can be observed in all the materials. However, the density of the microcracks in materials A3 and B3 are much higher than the other materials. The materials A2 and B2 are the ones having the lowest density of microcracks. The materials A3 and B3 are tested mechanically in cryogenic conditions (−54 °C), which probably have increased the brittleness of the material resulting in a high density of microcracks.

Since the materials A2 and B2 are tested at an elevated temperature, the material probably has plasticized before rupturing due to the mechanical load. This is probably the reason why the density of microcracks is less in those two materials. Materials A1 and B1 have a moderate density of microcracks between the materials tested in elevated and cryogenic conditions. Nonetheless, all the materials have shown the brittle fracture. The brittle fracture beach marks can be observed even in the materials A2 and B2. Although the material is tested in elevated temperature condition, the fracture, however, is brittle in nature. 

It will be intriguing to study the SEM analysis under the loading condition. It can provide more information about the nature of the damage and details on the surface morphology under loading.

## 4. Conclusions

In this work, a thermomechanical study on high-strength carbon/epoxy composite obtained by means of stitching and RFI was done. Three significantly different lay-ups have been tested and modeled. Experimental data suggest that tensile mechanical rigidity tends to increase when reducing temperature; this could be linked to the carbon fibers’ behavior regarding thermal stability. Thermal degradation kinetics on the same specimens were studied as well. The materials were tested mechanically in elevated temperature (71 °C), room temperature, and cryogenic conditions (−54 °C). The TG results show that the material degrades in a similar pattern; the onset temperature of degradation, maximum degradation temperature, and end set temperature remains the same for all the materials. However, the thermal kinetics results suggest otherwise. The activation energy of the material shows the degradation of materials tested in cryogenic conditions yields more char. Moreover, the weight percentage of carbon fiber in the segment of the material tested for thermal analysis plays a crucial role in varying the activation energy. The pre-exponential factor also has very similar values and can be related directly proportional to the activation energy. The optimum working temperature of material shows that the materials tested mechanically at elevated temperature have the lowest working temperature. The materials tested in room temperature have the best working temperature, thus suggesting the different environmental condition affects the thermal degradation of a material. Finally, the SEM morphology shows the density of microcracks. The materials tested in cryogenic conditions have a high density of microcracks when compared to materials tested in room temperature and elevated temperature. Thermal degradation kinetics can be very useful in recovering carbon fiber from CFRPs which are subjected to or used in different weathering conditions.

## Figures and Tables

**Table 1:** Denomination of materials and testing conditions.

Specimen Name	Resin Content	% Fiber Orientation Along [0°/±45°/90°]	No. of Plies	Environmental Testing Conditions
A1	39%	40(0°)/40(±45°)/20(90°)	10	RT
A2	39%	40(0°)/40(±45°)/20(90°)	10	H/W
A3	39%	40(0°)/40(±45°)/20(90°)	10	CT
B1	37.9%	40(0°)/40(±45°)/20(90°)	10	RT
B2	37.9%	40(0°)/40(±45°)/20(90°)	10	H/W
B3	37.9%	40(0°)/40(±45°)/20(90°)	10	CT
C1	40%	100(0°)/0(±45°)/0(90°)	14	RT
C2	40%	100(0°)/0(±45°)/0(90°)	14	H/W
C3	40%	100(0°)/0(±45°)/0(90°)	14	CT

**Table 2:** Mechanical test results for specimens tested under different environmental conditions.

Specimen Name	Environmental Condition	Ultimate Tensile Strength [MPa]	Young’s Modulus [GPa]
A1	RT	830 ± 98	71.13 ± 4.06
A2	H/W	1016 ± 63	85.73 ± 3.10
A3	CT	748 ± 31	137.60 ± 4.84
B1	RT	555 ± 18	43.57 ± 0.99
B2	H/W	605 ± 24	45.90 ± 0.99
B3	CT	509 ± 20	84.33 ± 2.09
C1	RT	1409 ± 95	277.90 ± 11.96
C2	H/W	789 ± 81	155.20 ± 6.99
C3	CT	767 ± 29	213.07 ± 9.59

**Table 3:** Pre-exponential factor of all the materials.

*α*	Pre-Exponential Factor (lnA)								
	A1	A2	A3	B1	B2	B3	C1	C2	C3
0.200	34.15	23.43	24.51	30.44	29.27	27.92	36.21	27.12	33.26
0.225	33.96	24.29	25.36	30.60	29.42	28.15	36.75	27.31	31.96
0.250	34.01	24.95	25.99	31.02	29.71	28.44	37.21	27.64	32.45
0.275	33.89	25.69	26.56	31.26	30.02	28.73	37.54	27.89	33.88
0.300	33.81	26.16	27.16	31.66	30.28	29.02	37.57	28.16	34.20
0.325	33.71	26.53	27.35	32.03	30.39	29.06	37.43	28.31	34.44
0.350	33.75	26.82	27.46	32.23	30.47	29.22	37.39	28.30	34.72
0.375	33.82	26.91	27.58	32.23	30.62	29.42	37.57	28.30	34.93
0.400	33.64	27.00	27.51	32.35	30.65	29.39	37.85	28.38	34.77
0.425	33.67	27.12	27.66	32.57	30.50	29.31	37.86	28.32	34.59
0.450	33.34	27.20	27.63	32.60	30.31	29.17	37.92	28.09	34.66
0.475	32.86	27.04	27.68	32.31	30.09	29.17	37.89	28.06	34.89
0.500	32.66	26.96	27.58	31.92	29.89	29.16	37.72	27.98	34.91
0.525	32.40	26.97	27.62	31.62	29.63	29.19	38.00	27.95	34.72
0.550	32.39	26.83	27.62	31.39	29.40	29.23	38.37	27.87	34.77
0.575	32.32	26.68	27.56	31.22	29.24	29.21	38.77	27.73	34.82
0.600	32.27	26.68	27.51	31.19	29.34	29.26	39.67	27.70	34.75
0.625	32.27	26.71	27.75	31.03	29.58	29.32	40.35	27.82	34.71
0.650	32.19	26.71	27.92	30.88	29.54	29.43	41.40	28.04	34.79
0.675	32.29	26.79	28.07	30.85	29.61	29.71	42.40	28.24	34.81
0.700	32.51	26.69	28.31	30.80	29.92	30.23	43.46	28.46	34.72
0.725	32.83	26.70	28.93	30.77	30.57	30.70	45.18	28.94	34.27
0.750	33.16	27.01	29.89	31.04	31.57	31.46	47.05	30.03	33.53
0.775	33.35	27.40	31.23	31.42	32.88	32.84	47.18	31.39	32.36
0.800	33.66	27.86	33.35	32.13	34.77	34.79	50.94	33.84	30.51

**Table 4:** Optimum working temperature for all the materials.

Lifetime (h)	Optimum Working Temperature (°C)								
	A1	A2	A3	B1	B2	B3	C1	C2	C3
20,000	152	115	116	146	134	142	170	127	161
10,000	160	124	125	154	143	150	178	135	169
5000	168	133	134	162	152	159	186	144	177
1000	189	156	156	183	173	180	206	166	197
100	222	193	194	217	209	214	237	202	230
10	261	238	238	257	250	255	273	245	267
1	306	292	292	303	299	302	314	296	310
